# Systems Pharmacology Investigation of Phytochemicals From *Scilla peruviana* L.: Integrated Gas Chromatography–Mass Spectrometry Profiling, Network Pharmacology, and Molecular Docking Reveal Potential Anti‐Inflammatory Mechanisms

**DOI:** 10.1002/open.70320

**Published:** 2026-10-21

**Authors:** Roukia Zatout, Ouided Benslama, Stefania Garzoli

**Affiliations:** ^1^ Department of Microbial Biotechnology Faculty of Natural and Life Science University of Blida 1 Blida Algeria; ^2^ Laboratory of Biological Engineering, Valorization and Innovation of Agri‐Food Products University of Larbi Ben M’hidi Oum El Bouaghi Algeria; ^3^ Research Unit of Environmental and Structural Molecular Chemistry University of Constantine (Mentouri) Constantine Algeria; ^4^ Department of Chemistry and Technologies of Drug Sapienza University Rome Italy

**Keywords:** anti‐inflammatory activity, fatty acids, molecular docking, network pharmacology, *Scilla peruviana* L., volatile compounds

## Abstract

This study aimed to characterize the volatile and fatty acid constituents of *S. peruviana* and investigate their potential anti‐inflammatory mechanisms. Volatile compounds were identified by headspace solid‐phase microextraction coupled with gas chromatography–mass spectrometry (HS‐SPME‐GC–MS), whereas fatty acids were analyzed by GC–MS following derivatization. The identified phytochemicals were subjected to target prediction, followed by protein–protein interaction (PPI), Gene Ontology (GO), Kyoto Encyclopedia of Genes and Genomes (KEGG) pathway enrichment analyses, and molecular docking. HS‐SPME‐GC–MS analysis identified 11 volatile compounds, predominantly hexahydrofarnesyl acetone (48.4%) and dihydroactinidiolide (23.5%), followed by dehydro‐β‐ionone (5.6%), 2‐methylbutanal (5.5%), and 3‐methylbutanal (5.1%). Fatty acid profiling revealed palmitic acid as the predominant fatty acid (47.7%), followed by α‐linolenic acid (29.9%), linoleic acid (11.6%), and stearic acid (10.8%). Network pharmacology identified 253 overlapping targets between phytochemical‐associated proteins and inflammation‐related genes, with TNF, IL1B, PTGS2, EGFR, STAT3, and PPARG emerging as the principal hub proteins. KEGG enrichment analysis revealed significant enrichment in inflammation‐associated pathways, including NF‐κB, IL‐17, NOD‐like receptor, and advanced glycation end‐products (AGE)‐receptor for advanced glycation end‐products (RAGE) signaling pathways. Molecular docking predicted interactions between the major phytochemicals and the selected targets, with palmitic acid showing the lowest predicted binding energy for COX‐2 (−7.0 kcal/mol), while hexahydrofarnesyl acetone and stearic acid showed the lowest predicted binding energies for PPARγ (−8.3 kcal/mol).

## Graphical abstract

This study aims to characterize the volatile and fatty acid constituents of Scilla peruviana and investigate their potential anti‐inflammatory mechanisms. Volatile compounds are identified by headspace solid‐phase microextraction (HS‐SPME) coupled with GC–MS, whereas fatty acids are analyzed by GC–MS following derivatization. The identified phytochemicals are subjected to target prediction, followed by protein–protein interaction, gene ontology, Kyoto Encyclopedia of Genes and Genomes (KEGG) pathway enrichment analyses, and molecular docking.

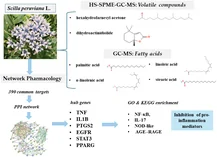

## 1 Introduction

Natural products remain an important source of bioactive molecules for drug discovery because of their remarkable chemical diversity and wide range of pharmacological activities [1, 2, 3]. Advances in metabolomics, analytical chemistry, and computational approaches have further strengthened the exploration of medicinal plants as reservoirs of compounds with therapeutic potential [4, 5, 6]. Among these resources, plants synthesize numerous secondary metabolites, including terpenoids, phenolics, flavonoids, alkaloids, and fatty acids, many of which exhibit antioxidant and anti‐inflammatory properties [7, 8].

Inflammation is a complex biological response that protects tissues against infection and injury; however, persistent activation of inflammatory pathways contributes to the development of chronic diseases such as cardiovascular disorders, diabetes, arthritis, neurodegenerative diseases, and cancer. Key mediators involved in these processes include TNF‐α, IL‐1β, COX‐2, iNOS, and the NF‐κB signaling pathway, which have become important targets for the development of safer anti‐inflammatory agents [9, 10]. Because medicinal plants contain multiple bioactive constituents acting on different molecular targets, network pharmacology has emerged as a valuable systems‐based approach to elucidate their mechanisms of action by integrating phytochemical profiling, target prediction, pathway enrichment, protein–protein interaction (PPI) analysis, and molecular docking [11, 12].

The genus *Scilla* L. (Asparagaceae) comprises bulbous perennial species widely distributed throughout the Mediterranean Basin and adjacent regions [13]. Several *Scilla* species have attracted considerable scientific interest owing to their rich phytochemical composition, particularly homoisoflavonoids, bufadienolides, saponins, and phenolic compounds, which have been reported to exhibit diverse biological activities, including antioxidant, antimicrobial, anticancer, and anti‐inflammatory effects [14, 15]. However, previous investigations have primarily focused on nonvolatile secondary metabolites, whereas the volatile constituents and lipid composition of *Scilla* species remain poorly characterized.

Among them, *Scilla peruviana* L. has received relatively limited phytochemical and pharmacological attention. Although the occurrence of fatty acids in higher plants is well established, their contribution to the biological properties of *S. peruviana* has not been comprehensively investigated. Fatty acids are essential components of plant membranes and play important roles in cellular metabolism and biological regulation [16]. Moreover, the coexistence of saturated and unsaturated fatty acids may contribute to the biological potential of plant extracts through complementary or synergistic effects [17].

Therefore, the present study aimed to comprehensively characterize the volatile phytochemical profile of *Scilla peruviana* L. using gas chromatography–mass spectrometry (GC–MS) and to investigate its potential anti‐inflammatory mechanisms through an integrated computational approach. Specifically, network pharmacology, PPI analysis, functional enrichment, and molecular docking were employed to identify key molecular targets, signaling pathways, and phytochemical constituents potentially associated with the predicted anti‐inflammatory effects of *S. peruviana*. This integrated strategy was intended to provide mechanistic insights into the potential biological relevance of the identified constituents and to establish a computational framework that can guide and support future experimental validation of the proposed anti‐inflammatory activity.

## 2 Materials and Methods

### 2.1 Plant Material and Identification

Aerial parts of *Scilla peruviana* L. were collected from Jebel El Ouahch Forest, Constantine, northeastern Algeria, during the flowering season in April 2025 (Figure 1). Jebel El Ouahch Forest is one of the main forest ecosystems in northeastern Algeria and is characterized by a Mediterranean climate with diverse vegetation, as previously described by Zatout et al. [18]. The species was identified based on its diagnostic morphological characteristics, including a large ovoid bulb, a basal rosette of linear‐lanceolate leaves, and a dense conical inflorescence composed of numerous blue star‐shaped flowers. The scientific name and current taxonomic status were verified using Plants of the World Online (Royal Botanic Gardens, Kew). The collected plant material was cleaned, air‐dried at room temperature, ground into a fine powder, and stored in airtight containers until further analysis.

**FIGURE 1:**
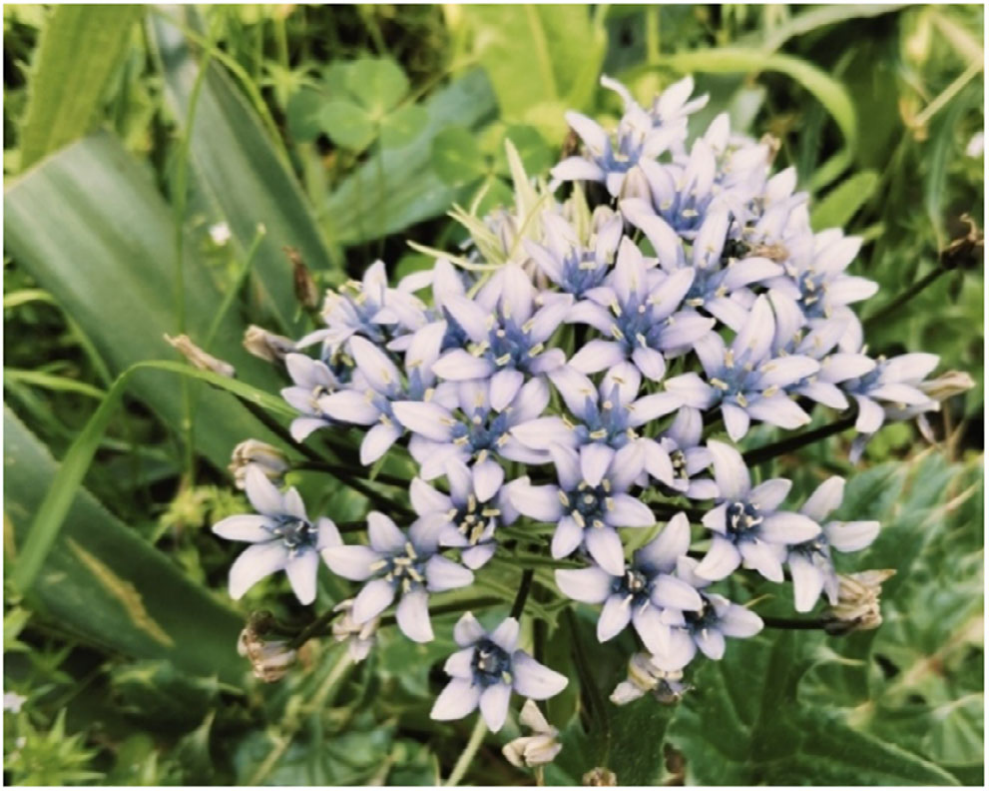
Morphological characteristics of *Scilla peruviana* L.

### 2.2 Volatile Profiling of *Scilla peruviana* L.

The volatile constituents of *S*. *peruviana* were characterized using headspace solid‐phase microextraction coupled with GC–MS (HS‐SPME–GC–MS) (Clarus 500, PerkinElmer, USA). Approximately 0.5 g of the dried powdered sample was transferred into a sealed headspace vial (4 mL) and equilibrated at 60 °C for 20 min before extraction. Volatile compounds were adsorbed onto a DVB/CAR/PDMS SPME fiber and subsequently desorbed into a GC injector at 250 °C in the splitless mode The GC oven was equipped with a VF‐5 capillary column (60 m × 0.32 mm × 1.0 μm). The oven temperature was initially maintained at 45 °C and then increased to 220 °C at a rate of 6 °C min^−1^, followed by a final hold of 15 min. Helium was used as carrier gas at a constant rate of 1 mL/min. MS scans were recorded within the range of 35–450 m/z using EI ionization (energy 70 eV). Compound identification was achieved by comparing the obtained mass spectra with the NIST and Wiley spectral libraries and by calculating linear retention indices using a homologous series of *n*‐alkanes (C_8_–C_25_). The relative abundance of each volatile constituent was expressed as the percentage of its chromatographic peak area with respect to total area. All analyses were carried out in triplicate.

### 2.3 Fatty Acids Profiling of *Scilla peruviana* L.

The fatty acid profile of *S. peruviana* was determined by GC–MS following lipid extraction and conversion into fatty acid methyl esters (FAMEs). Lipids were extracted using a chloroform:methanol mixture (2:1, v/v), and the resulting extracts were transmethylated with boron trifluoride in methanol (BF_3_–methanol). The FAMEs were analyzed on a Clarus 500 GC–MS system (PerkinElmer, USA). Chromatographic separation was carried out using the same column (VF‐5) while the injector was maintained at 280 °C, and the oven temperature was programed from 170 to 260 °C at a heating rate of 3 °C min^−1^ and held at the final temperature for 15 min. Fatty acids were identified by comparison of their mass spectra and linear retention indices with reference databases. Their relative contents were calculated as percentages of the total chromatographic peak area. All analyses were carried out in triplicate.

### 2.4 Network Pharmacology Analysis

#### 2.4.1 Prediction of Phytochemical‐Related Targets

The chemical structures of the volatile compounds identified by GC–MS analysis of *Scilla peruviana* L. (syn. *Oncostema peruviana* (L.) Speta) were retrieved from the PubChem database (https://pubchem.ncbi.nlm.nih.gov/, accessed June 14, 2026) in SMILES format. The SMILES strings of each compound were individually submitted to the SwissTargetPrediction web server (http://www.swisstargetprediction.ch/, accessed June 14, 2026) to predict their potential human protein targets.

SwissTargetPrediction predicts ligand–target interactions based on the structural similarity of query molecules to experimentally validated ligands using a combination of two‐dimensional (2D) and three‐dimensional (3D) similarity measures. For all predictions, the target organism was restricted to *Homo sapiens*, and only targets with a prediction probability ≥ 0.1 were retained for subsequent analyses. This more stringent threshold was applied to reduce the inclusion of low‐confidence target predictions. To assess the robustness of the network pharmacology findings, the target‐selection procedure was compared with the initial analysis performed using probability > 0, and the resulting target sets and hub genes were examined for consistency [19].

The target lists obtained for each phytochemical were merged into a single dataset, and duplicate entries were removed to generate a nonredundant list of compound‐related targets for downstream network pharmacology analyses.

A sensitivity analysis was performed to evaluate the effect of the SwissTargetPrediction probability threshold on the network pharmacology results. The initial target dataset obtained using probability > 0 was compared with the revised dataset generated using the more stringent probability ≥ 0.1 criterion. The number of predicted targets, the number of overlapping inflammation‐associated targets, and the resulting hub genes were compared between the two analyses.

#### 2.4.2 Collection of Inflammation‐Related Targets

Inflammation‐associated genes were collected from three publicly available databases, namely, GeneCards (https://www.genecards.org/, accessed June 14, 2026), Online Mendelian Inheritance in Man (OMIM, https://www.omim.org/, accessed June 14, 2026), and DisGeNET (https://www.disgenet.org/, accessed June 14, 2026). The keyword “inflammation” was used independently in each database to retrieve genes associated with inflammatory diseases and immune‐related biological processes (BP).

The retrieved gene lists from the three databases were integrated into a single dataset. Redundant entries originating from overlapping records were identified and removed using Microsoft Excel to generate a comprehensive, nonredundant list of inflammation‐related genes. This integrated dataset was subsequently used for the identification of potential therapeutic targets shared with the predicted targets of *S. peruviana* phytochemicals.

#### 2.4.3 Identification of Common Therapeutic Targets

To identify the potential therapeutic targets of *S. peruviana* phytochemicals against inflammation, the predicted compound‐related targets were intersected with the inflammation‐associated gene set. The overlap analysis was performed using the online Venn diagram platform Bioinformatics & Evolutionary Genomics (https://bioinformatics.psb.ugent.be/webtools/Venn/) [20].

The shared targets between the two datasets were considered inflammation‐associated candidate targets potentially linked to the biological effects of *S. peruviana* phytochemicals. These overlapping genes were subsequently used for PPI network construction, hub gene identification, functional enrichment analyses (Gene Ontology (GO) and KEGG pathway), and molecular docking studies.

#### 2.4.4 PPI Network Construction

The overlapping targets identified between *S. peruviana* phytochemical‐related targets and inflammation‐associated genes were imported into the STRING database (Search Tool for the Retrieval of Interacting Genes/Proteins, https://string‐db.org/, accessed June 16, 2026) to investigate their PPI network. The analysis was performed by selecting *Homo sapiens* as the target organism, with a high‐confidence interaction score threshold of 0.700. Disconnected nodes were excluded from the network to improve the reliability and interpretability of the interaction analysis [21].

The generated PPI network was exported in TSV format and subsequently imported into Cytoscape software (version 3.10.4) for network visualization and topological analysis. The resulting network served as the basis for identifying highly connected proteins (hub genes) that may play essential roles in the anti‐inflammatory mechanisms of *S. peruviana* phytochemicals.

#### 2.4.5 Identification of Hub Genes

To identify the key proteins involved in the anti‐inflammatory activity of *S. peruviana* phytochemicals, the PPI network was analyzed using the CytoHubba plugin implemented in Cytoscape (version 3.10.4) [22].

Among the available topological algorithms, the degree method was employed to evaluate the connectivity of each node by calculating the number of direct interactions with neighboring proteins. Proteins exhibiting higher degree values were considered to play central regulatory roles within the PPI network and were therefore identified as hub genes. The top 10 ranked genes were selected for further analysis and were subsequently used to identify key anti‐inflammatory targets and to support the selection of proteins for molecular docking studies.

#### 2.4.6 Functional Enrichment Analysis

To investigate the biological functions and potential molecular mechanisms associated with the inflammation‐related targets of *S. peruviana* phytochemicals, functional enrichment analyses were performed using the ShinyGO v0.81 web server (http://bioinformatics.sdstate.edu/go/, accessed June 20, 2026). The list of overlapping therapeutic targets was uploaded to ShinyGO, with *Homo sapiens* as the reference organism.

GO enrichment analysis was conducted to investigate significantly enriched BP, molecular functions (MF), and cellular components (CC). In addition, Kyoto Encyclopedia of Genes and Genomes (KEGG) pathway enrichment analysis was performed to identify the principal signaling pathways potentially involved in the potential anti‐inflammatory mechanisms associated with the identified phytochemicals.

Enrichment significance was assessed using the hypergeometric test, followed by false discovery rate (FDR) correction based on the Benjamini–Hochberg method. Terms with an adjusted *p*‐value (FDR) < 0.05 were considered statistically significant. The enriched GO terms and KEGG pathways were ranked according to their statistical significance and visualized using bubble plots generated by ShinyGO [23].

#### 2.4.7 Molecular Docking Analysis

The 3D chemical structures of the selected phytochemicals identified from *S. peruviana* were retrieved from the PubChem database (https://pubchem.ncbi.nlm.nih.gov/) in the Structure Data File (SDF) format. The ligand structures were energy‐minimized and converted into the PDBQT format using AutoDock Tools (version 1.5.7) prior to molecular docking [24]. Hydrogen atoms and partial atomic charges were assigned during ligand preparation, and the resulting structures were used for subsequent docking calculations.

The crystallographic structures of the selected hub proteins were retrieved from the Protein Data Bank (PDB, https://www.rcsb.org/). The selected targets included tumor necrosis factor‐alpha (TNF‐α; PDB ID: 2AZ5), cyclooxygenase‐2 (COX‐2/PTGS2; PDB ID: 5IKR), peroxisome proliferator‐activated receptor gamma (PPARγ; PDB ID: 4EMA), signal transducer and activator of transcription 3 (STAT3; PDB ID: 6NJS), and epidermal growth factor receptor (EGFR; PDB ID: 1M17).

Protein preparation was performed using AutoDock Tools. Water molecules, buffer ions, and other nonessential heteroatoms were removed from each crystal structure, while the cocrystallized ligand was extracted and retained separately for validation of the docking protocol through redocking. Polar hydrogen atoms were added, Kollman united‐atom charges were assigned, and nonpolar hydrogen atoms were merged. The prepared protein structures were subsequently converted into the PDBQT format for molecular docking [25].

Molecular docking simulations were carried out using AutoDock Vina, version 1.2.7. For each target, the docking grid was centered on the binding pocket occupied by the corresponding cocrystallized ligand. The grid‐box center coordinates (*x*, *y*, and *z*) and dimensions (size_*x*, size_*y*, and size_*z*) were defined individually for each protein as follows: TNF‐α (2AZ5), center = (−19.4096, 74.65075, 33.84955) Å and size = (23.2797, 23.2797, 23.2797) Å; COX‐2 (5IKR), center = (38.96122, 2.35269, 61.50347) Å and size = (22.3002, 22.3002, 22.3002) Å; PPARγ (4EMA), center = (14.7844, 7.0820, 44.50356) Å and size = (23.3041, 23.3041, 23.3041) Å; STAT3 (6NJS), center = (13.02832, 55.63712, 0.25017) Å and size = (31.6749, 31.6749, 31.6749) Å; and EGFR (1M17), center = (22.01369, 0.25283, 52.79403) Å and size = (26.6117, 26.6117, 26.6117) Å. The exhaustiveness parameter was set to 32, and up to 9 binding poses were generated for each ligand. All docking calculations were performed using the AutoDock Vina scoring function, and the binding affinity of each ligand was expressed as the predicted binding energy (kcal/mol) [26].

To validate the docking protocol, the cocrystallized ligand of each protein was extracted and redocked into its original binding site using the same docking parameters applied to the phytochemicals. The accuracy of the docking procedure was assessed by calculating the root‐mean‐square deviation (RMSD) between the experimentally determined and redocked ligand conformations. The obtained RMSD values were 1.501 Å for TNF‐α (PDB ID: 2AZ5), 1.845 Å for COX‐2 (PDB ID: 5IKR), 1.275 Å for PPARγ (PDB ID: 4EMA), 1.366 Å for STAT3 (PDB ID: 6NJS), and 1.117 Å for EGFR (PDB ID: 1M17), respectively. All RMSD values were below 2.0 Å, indicating an acceptable reproduction of the experimentally observed binding modes and supporting the suitability of the docking protocol for subsequent analyses [27, 28].

The binding poses and intermolecular interactions, including hydrogen bonds, hydrophobic interactions, electrostatic interactions, and π‐related contacts, were analyzed and visualized using the BIOVIA Discovery Studio Visualizer (version 2024, Dassault Systèmes, San Diego, CA, USA) [29].

## 3 Results and Discussion

### 3.1 Volatile Profiling of Fruits and Stem‐Leaves of *Scilla peruviana* L.

The volatile profile of *S. peruviana* obtained by HS‐SPME‐GC–MS was characterized by the predominance of oxygenated terpenoid derivatives and carotenoid‐derived norisoprenoids (Table 1 and Figure 2). Among the 11 identified volatile compounds, hexahydrofarnesyl acetone was the major constituent (48.4%), followed by dihydroactinidiolide (23.5%). Other constituents were detected in considerably lower amounts, including dehydro‐β‐ionone, 2‐methylbutanal, 3‐methylbutanal, 2‐phenyl‐5‐methyl‐2‐hexenal, megastigmatrienone, myrtenal, 3‐ethyl‐4‐methyl‐1H‐pyrrole‐2,5‐dione, pinocarvone, and 2‐decanone.

**TABLE 1:** Volatile composition of *Scilla peruviana* L. (percentage mean values ± SD).

N.	Component^a^	LRI^calc^ b	LRI^lit^ c	%
1	3‐Methyl, butanal	648	650	5.1 ± 0.20
2	2‐Methyl, butanal	670	672	5.5 ± 0.14
3	Pinocarvone	1138	1140	0.7 ± 0.03
4	2‐Decanone	1170	1171	0.7 ± 0.04
5	Myrtenal	1177	1176	1.9 ± 0.08
6	3‐Ethyl‐4‐methyl‐1H‐pyrrole‐2,5‐dione	1261	1265	1.8 ± 0.06
7	Dehydro‐β‐ionone	1432	1436	5.6 ± 0.11
8	Megastigmatrienone	1472	1475	3.5 ± 0.08
9	2‐Phenyl‐5‐methyl‐2‐hexenal	1488	1486	3.4 ± 0.16
10	Dihydroactinidiolide	1521	1525	23.5 ± 1.22
11	Hexaydrofarnesyl acetone	1820	1822	48.4 ± 3.12
	Total	100.0		

a The components are reported according to their elution order on apolar column.

b Linear retention indices measured on apolar column.

c Linear retention indices from literature.

**FIGURE 2:**
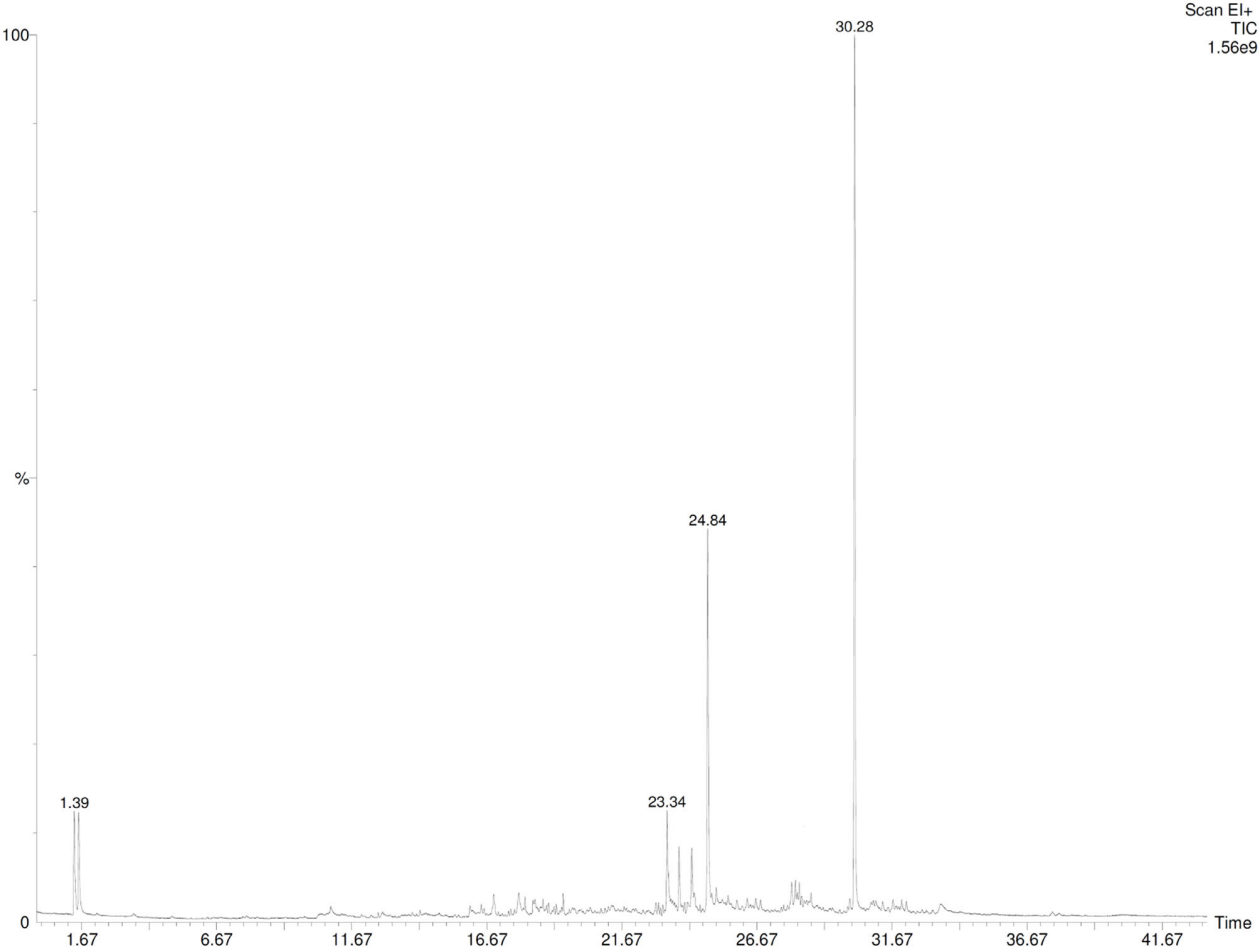
HS‐SPME‐GC–MS chromatogram of *Scilla peruviana* L.

To the best of our knowledge, information regarding the volatile constituents of *S. peruviana* remains extremely limited. Previous phytochemical studies have mainly focused on nonvolatile constituents, particularly lanostane‐type triterpene glycosides and homoisoflavonoids isolated from the bulbs, whereas their volatile metabolome has received little attention. Consequently, the present work expands the phytochemical knowledge of this Mediterranean species by providing the first detailed characterization of its volatile fraction [30, 31, 32].

The predominance of hexahydrofarnesyl acetone is particularly noteworthy. This compound has been reported as a common constituent of several medicinal and aromatic plants and has been associated with antioxidant, antimicrobial, insecticidal, and anti‐inflammatory properties, suggesting that it may contribute significantly to the biological activity of the volatile extract [33, 34, 35]. Likewise, the abundance of dihydroactinidiolide, a carotenoid‐derived norisoprenoid, together with dehydro‐β‐ionone and megastigmatrienone, indicates that carotenoid oxidative degradation represents an important metabolic pathway in *S. peruviana*. Norisoprenoids have attracted increasing interest because of their biological activities, particularly their antioxidant and anti‐inflammatory potential. Although present in relatively low amounts, compounds such as myrtenal and pinocarvone may also contribute to the overall biological activity of the volatile fraction through additive or synergistic effects. The chemical structures of the bioactive volatiles are reported in Figure 3.

**FIGURE 3:**
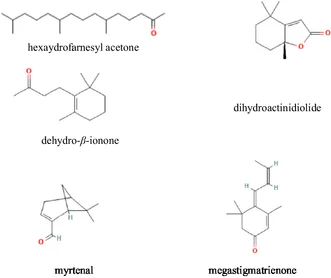
Structure of the bioactive volatiles.

Similarly, 2‐methylbutanal and 3‐methylbutanal are volatile aldehydes derived from branched‐chain amino acid metabolism and are frequently detected in plant volatilomes, where they contribute to the aroma and may participate in ecological interactions.

Overall, the volatile composition of *S. peruviana* differs markedly from the phytochemical profile previously described for this species, which has largely emphasized nonvolatile metabolites. The predominance of biologically active oxygenated terpenoid derivatives and carotenoid‐derived norisoprenoids provides a plausible chemical basis for the anti‐inflammatory mechanisms predicted by the subsequent network pharmacology and molecular docking analyses. These findings broaden the current phytochemical knowledge of S. peruviana and support its potential as a promising source of bioactive natural products deserving further pharmacological investigation [36].

### 3.2 Fatty Acid Profiling of *Scilla peruviana* L.

The fatty acid composition of *S. peruviana* was dominated by palmitic acid (47.7%), followed by α‐linolenic acid (29.9%), linoleic acid (11.6%), and stearic acid (10.8%) (Table 2).

**TABLE 2:** Fatty acids content of of *Scilla peruviana* L. (percentage mean values ± standard deviation).

N.	Fatty acids	LRI^calc^	LRI^lit^	%
1	Palmitic acid, C16:0	1971	1973	47.7 ± 3.55
2	Linoleic acid, C18:2*n*6	2150	2152	11.6 ± 1.25
3	*α*‐Linolenic acid C18:3*n*2	2160	2158	29.9 ± 2.11
4	Staeric acid, C18:0	2175	2178	10.8 ± 0.67
	Sum			100.0
	Saturated FAs			58.5
	Unsaturated FAs			41.5

*Note*: The components are reported according to their elution order on apolar column (VF‐5 ms); linear retention indices measured on apolar column; linear retention indices from literature.

This profile indicates that the lipid fraction contains both saturated and unsaturated fatty acids, with saturated fatty acids representing the largest proportion. Similar fatty acids have been reported in a wide range of plant species, where they contribute to the membrane structure, energy storage, and several physiological processes [17].

Among the identified compounds, palmitic acid was the most abundant. As one of the major saturated fatty acids in higher plants, it is an essential component of membrane lipids and has frequently been identified in medicinal plants. Although palmitic acid is mainly considered a structural lipid, it has also been reported to contribute to the biological activities of plant extracts, particularly when acting together with other metabolites rather than as an isolated compound [37, 38, 39].

The relatively high content of α‐linolenic acid is noteworthy because this essential ω‐3 polyunsaturated fatty acid has been widely associated with beneficial biological effects. Previous studies have demonstrated that α‐linolenic acid can regulate inflammatory responses by modulating cytokine production and inflammatory signaling pathways, including NF‐κB, thereby contributing to the maintenance of immune homeostasis [10, 16].

Linoleic acid, the main ω‐6 fatty acid detected in the present study, is another essential fatty acid involved in membrane integrity and cellular signaling. In plants, it serves as a precursor for several oxylipins that participate in defense responses, while in mammals, it contributes to the synthesis of bioactive lipid mediators involved in normal physiological processes [40, 41].

Although stearic acid accounted for a smaller proportion of the total fatty acids, it is commonly found in plant lipids and contributes to membrane stability and energy metabolism. The coexistence of saturated fatty acids with relatively high levels of polyunsaturated fatty acids suggests that *S. peruviana* possesses a chemically balanced lipid profile that may contribute to its overall biological properties [39, 42].

Overall, the predominance of palmitic acid, together with the considerable amounts of α‐linolenic and linoleic acids, expands the current phytochemical knowledge of *S. peruviana*. These findings complement the volatile metabolite profile reported in the present study and provide additional information on the chemical composition of this species, which may be useful for future investigations exploring its biological and pharmacological potential.

### 3.3 Molecular Docking

#### 3.3.1 Identification of Overlapping Therapeutic Targets Between *Scilla peruviana* L. *P*hytochemicals and Inflammation‐Associated Genes

To investigate the potential molecular associations of *S. peruviana* phytochemicals with inflammation‐related targets, the predicted phytochemical targets were intersected with inflammation‐associated genes collected from GeneCards, OMIM, and DisGeNET. Using the more stringent SwissTargetPrediction probability threshold (probability ≥ 0.1), 254 unique human protein targets were identified for the selected phytochemicals, whereas 23,438 inflammation‐associated genes were retrieved after integration of the three disease databases. The overlap analysis identified 253 common targets, with only one target occurring exclusively among the predicted phytochemical‐associated proteins (Figure 4). Thus, 99.6% of the predicted phytochemical targets were represented within the inflammation‐associated gene set. Importantly, the revised analysis substantially reduced the number of retained phytochemical‐associated targets compared with the initial probability > 0 analysis (391 targets) while preserving the principal hub genes identified in the downstream network analysis.

**FIGURE 4:**
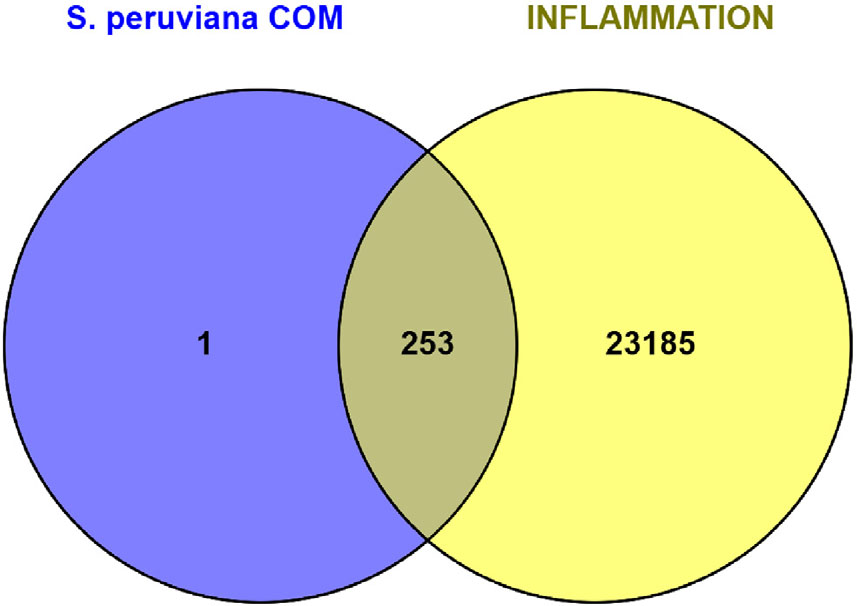
Venn diagram illustrating the overlap between the predicted targets of *Scilla peruviana* L. phytochemicals obtained using a SwissTargetPrediction probability threshold of ≥0.1 and inflammation‐associated genes. A total of 254 phytochemical‐associated targets and 23,438 inflammation‐associated genes were identified, with 253 targets shared between the two datasets.

The identification of such a large number of overlapping targets suggests that the phytochemical constituents of *S. peruviana* may exert their anti‐inflammatory activity through a multitarget mode of action. Unlike conventional single‐target drugs, phytochemicals are known to simultaneously regulate numerous proteins involved in interconnected BP, thereby producing synergistic pharmacological effects [11]. This characteristic represents one of the fundamental concepts of network pharmacology and is particularly relevant for complex diseases such as inflammation, where multiple signaling cascades and regulatory proteins contribute to disease initiation and progression [43].

The broad overlap observed between phytochemical targets and inflammation‐associated genes also indicates that the bioactive constituents of *S. peruviana* may influence diverse biological functions implicated in inflammatory responses, including cytokine production, immune regulation, intracellular signal transduction, oxidative stress, apoptosis, and cell proliferation. These findings support the hypothesis that the anti‐inflammatory activity of *S. peruviana* is mediated through coordinated modulation of multiple molecular targets rather than a single signaling pathway.

To further characterize the functional relationships among these overlapping therapeutic targets and to identify the key regulatory proteins involved in the anti‐inflammatory network, a PPI network was subsequently constructed using the STRING database.

#### 3.3.2 PPI Network Analysis

To investigate the functional relationships among the 253 overlapping therapeutic targets, a PPI network was constructed using the STRING database and subsequently visualized in Cytoscape. As illustrated in Figure 5, the resulting network revealed an extensive interaction pattern among the identified proteins, indicating that the majority of the overlapping targets are functionally interconnected rather than acting independently.

**FIGURE 5:**
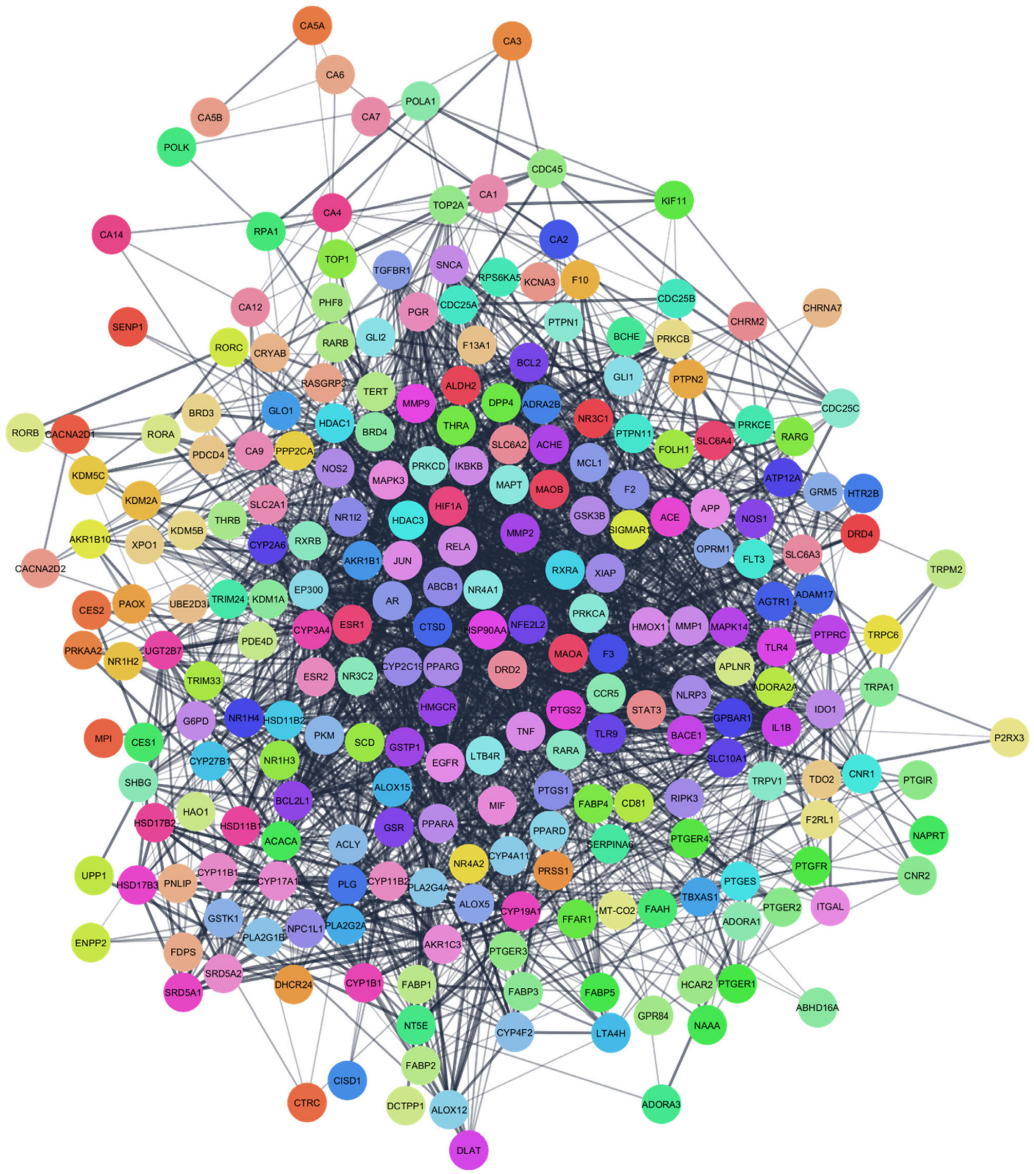
Protein–protein interaction (PPI) network of the 253 overlapping therapeutic targets identified between *Scilla peruviana* L. phytochemical‐associated proteins and inflammation‐associated genes. The network was constructed using STRING and visualized in Cytoscape. Nodes represent proteins, whereas edges indicate experimentally validated or predicted functional associations.

The high degree of connectivity observed within the PPI network suggests that the predicted targets participate in highly coordinated BP involved in the regulation of inflammation. Since inflammatory responses arise from the integration of multiple signaling pathways rather than the activation of isolated proteins, densely interconnected interaction networks are expected to characterize inflammatory diseases. Consequently, perturbation of a limited number of central regulatory proteins may influence numerous downstream signaling cascades, thereby contributing to the therapeutic effects of bioactive phytochemicals.

From a systems pharmacology perspective, the observed interaction network supports the hypothesis that *S. peruviana* phytochemicals exert their anti‐inflammatory activity through the simultaneous modulation of multiple molecular targets. Such a multitarget pharmacological profile is consistent with the complex chemical composition of medicinal plants and provides a mechanistic basis for their broad biological activities [12].

Considering that not all proteins contribute equally to network organization, topological analysis was subsequently performed to identify the most influential nodes (hub genes), which may represent key molecular regulators of the anti‐inflammatory effects of *S. peruviana* phytochemicals.

#### 3.3.3 Identification of Hub Genes

To identify the most influential proteins within the inflammatory interaction network, topological analysis was performed. The ten highest‐ranked hub genes are presented in Figure 6A,B. Among them, TNF exhibited the highest connectivity (degree = 158), followed by IL1B (143), EGFR (131), SRC (128), PTGS2 (123), STAT3 (121), ESR1 (119), BCL2 (119), PPARG (118), and HSP90AA1 (117), indicating their central regulatory roles within the inflammatory protein interaction network.

**FIGURE 6:**
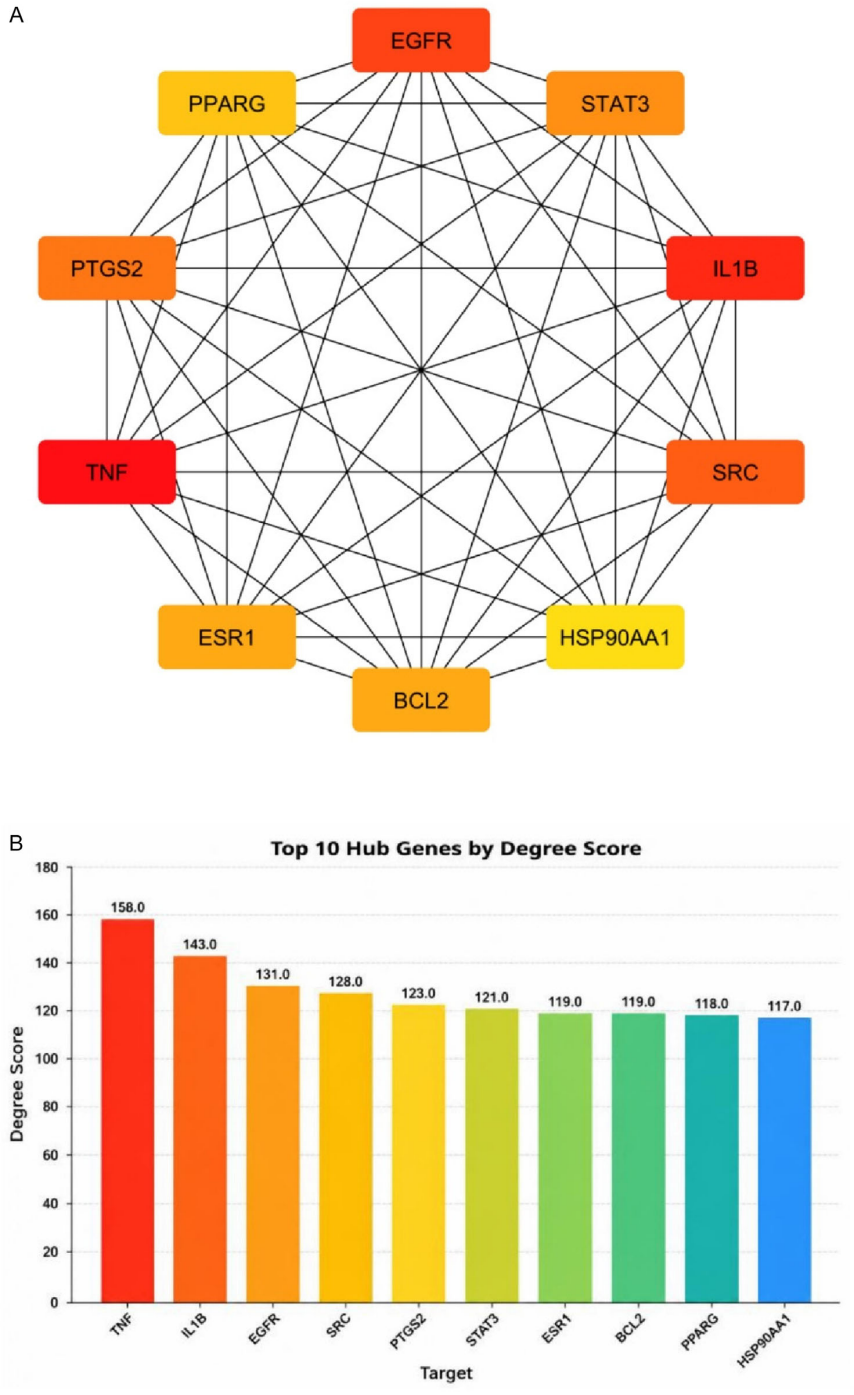
Identification of the top 10 hub genes within the protein–protein interaction network. (A) Subnetwork of the 10 highest‐ranked hub genes extracted from the PPI network. Node color intensity reflects the relative degree value, with darker red indicating higher connectivity. (B) Degree scores of the top 10 hub genes, highlighting TNF, IL1B, EGFR, SRC, PTGS2, STAT3, ESR1, BCL2, PPARG, and HSP90AA1 as the principal regulatory nodes potentially involved in the anti‐inflammatory activity of *Scilla peruviana* L. phytochemicals.

The predominance of TNF and IL1B among the identified hub genes underscores the pivotal role of pro‐inflammatory cytokines in orchestrating inflammatory responses. TNF‐α is widely recognized as a master regulator of inflammation, capable of activating multiple downstream signaling pathways, including NF‐κB, MAPK, and JAK/STAT, thereby promoting the expression of numerous inflammatory mediators [44, 45]. Similarly, IL1B functions as a key initiator of innate immune responses by stimulating leukocyte recruitment, cytokine secretion, and amplification of inflammatory cascades [46]. Their high connectivity suggests that modulation of these cytokines may exert widespread regulatory effects on the inflammatory network.

In addition to inflammatory cytokines, several signaling regulators, including EGFR, SRC, and STAT3, were identified as highly connected nodes. These proteins play fundamental roles in intracellular signal transduction and regulate diverse BP such as cell proliferation, differentiation, immune activation, and cytokine‐mediated signaling [47]. Their simultaneous identification indicates that the anti‐inflammatory activity of *S. peruviana* phytochemicals may involve coordinated regulation of multiple signaling pathways rather than inhibition of a single molecular target.

Interestingly, PTGS2 (COX‐2) and PPARG, two well‐established pharmacological targets in inflammatory diseases, were also among the top‐ranked hub genes. PTGS2 catalyzes the synthesis of pro‐inflammatory prostaglandins and represents the primary target of nonsteroidal anti‐inflammatory drugs (NSAIDs) [48, 49], whereas PPARG acts as a nuclear receptor with potent anti‐inflammatory properties by negatively regulating NF‐κB‐dependent transcription [50]. The presence of both proteins among the principal hub genes suggests that *S. peruviana* phytochemicals may simultaneously modulate inflammatory mediator production and transcriptional regulation of inflammatory responses.

Moreover, ESR1, BCL2, and HSP90AA1 were identified as additional central components of the interaction network. These proteins are involved in hormone‐mediated immune regulation, apoptosis [51], protein folding [52], and maintenance of cellular homeostasis, highlighting the complexity of the molecular mechanisms potentially underlying the anti‐inflammatory effects of the investigated phytochemicals.

Collectively, the hub gene analysis indicates that the anti‐inflammatory activity of *S. peruviana* is likely mediated through the coordinated regulation of cytokine signaling, inflammatory mediator biosynthesis, intracellular signal transduction, and cell survival pathways.

#### 3.3.4 Functional Enrichment Analysis

To further elucidate the biological functions and molecular mechanisms associated with the identified therapeutic targets, GO and KEGG enrichment analyses were performed using ShinyGO. The significantly enriched GO terms and KEGG pathways are presented in Figure 7A–D.

**FIGURE 7:**
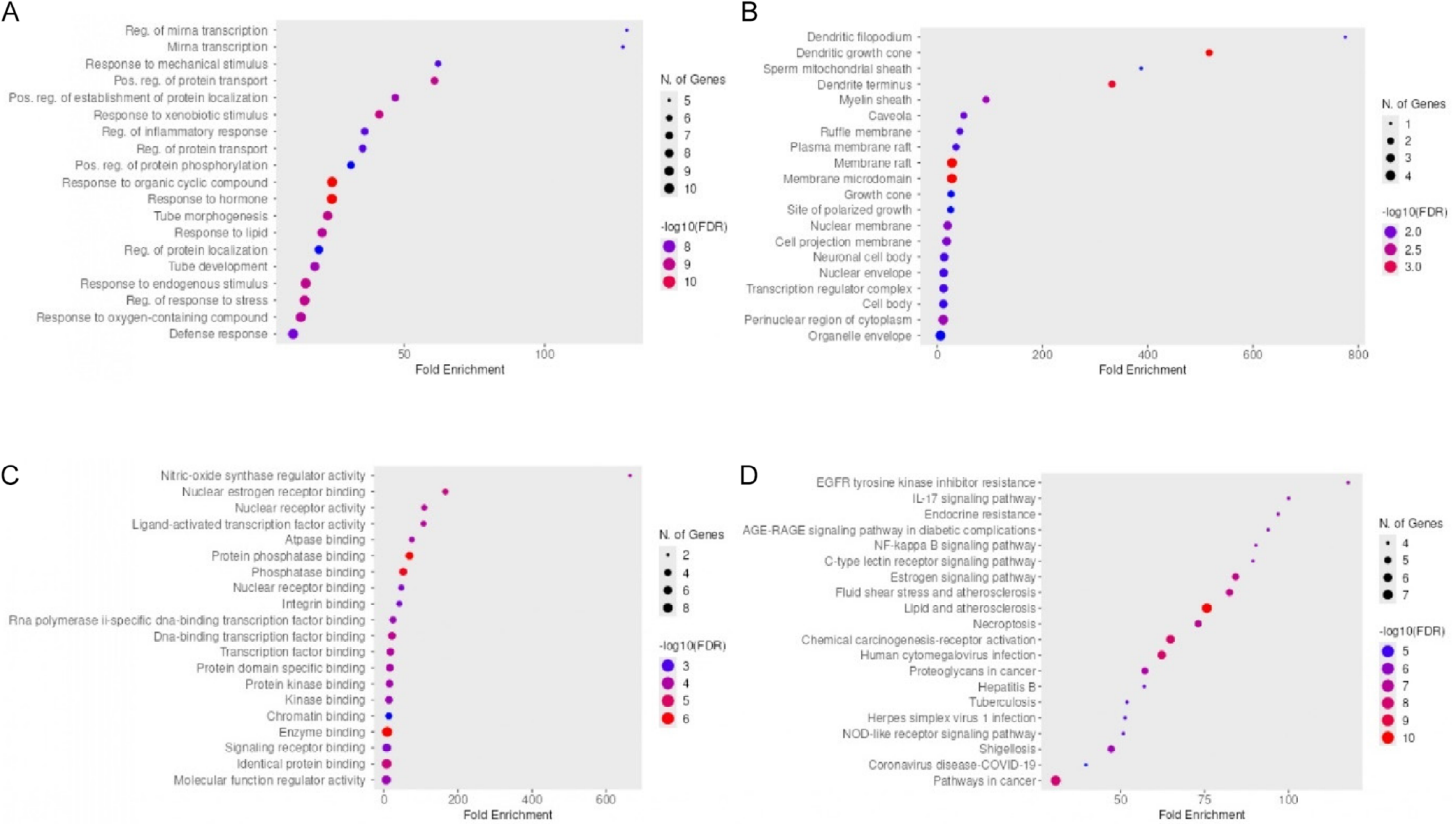
Functional enrichment analysis of the 253 overlapping therapeutic targets identified for *Scilla peruviana* L. phytochemicals. (A) Gene ontology (GO) biological process (BP) enrichment analysis, (B) GO cellular component (CC) enrichment analysis, (C) GO molecular function (MF) enrichment analysis, and (D) Kyoto Encyclopedia of Genes and Genomes (KEGG) pathway enrichment analysis. Bubble size represents the number of genes enriched in each term, while bubble color indicates the enrichment significance expressed as −log_10_ (false discovery rate, FDR). The *x*‐axis represents the fold enrichment of each GO term or KEGG pathway.

GO BP enrichment analysis (Figure 7A) revealed that the overlapping therapeutic targets were predominantly associated with the regulation of inflammatory and cellular responses, including regulation of inflammatory response, response to stress, response to oxygen‐containing compounds, response to lipid, protein phosphorylation, regulation of protein localization, and protein transport. These BP collectively indicate that *S. peruviana* phytochemicals may influence multiple aspects of the inflammatory cascade, ranging from immune activation and oxidative stress responses to intracellular signaling and cellular homeostasis.

The CC analysis (Figure 7B) demonstrated that the enriched targets were mainly localized in membrane‐associated compartments, including membrane rafts, plasma membrane rafts, membrane microdomains, caveolae, nuclear membrane, and transcription regulator complexes. Since membrane microdomains act as signaling platforms for numerous inflammatory receptors and kinases, these findings suggest that the predicted targets participate in the spatial organization of inflammatory signaling events.

GO MF analysis (Figure 7C) indicated significant enrichment in protein kinase binding, phosphatase binding, nuclear receptor binding, transcription factor binding, enzyme binding, ATPase binding, and ligand‐activated transcription factor activity. These MF highlight the ability of the identified targets to regulate intracellular signal transduction, transcriptional control, and enzyme‐mediated inflammatory processes.

KEGG pathway enrichment analysis (Figure 7D) further demonstrated that the overlapping targets were significantly involved in several inflammation‐related signaling pathways, including the IL‐17 signaling pathway, NF‐κB signaling pathway, C‐type lectin receptor signaling pathway, advanced glycation end‐products (AGE)‐receptor for advanced glycation end‐products (RAGE) signaling pathway in diabetic complications, and estrogen signaling pathway. In addition, pathways associated with immune regulation, infectious diseases, and cancer, such as NOD‐like receptor signaling, human cytomegalovirus infection, hepatitis B, herpes simplex virus 1 infection, and pathways in cancer, were also significantly enriched. The enrichment of these pathways reflects the extensive crosstalk between inflammation, immune regulation, and cellular stress responses. These findings indicate that the identified phytochemical‐associated targets are connected to multiple inflammation‐related BP and signaling pathways, providing hypotheses for further investigation of the potential biological effects of *S. peruviana* constituents.

#### 3.3.5 Molecular Docking Analysis

To further explore the potential molecular interactions between *S. peruviana* phytochemicals and inflammation‐associated hub proteins, molecular docking analysis was performed against five key proteins identified through network pharmacology, namely, TNF‐α, PTGS2/COX‐2, PPARγ, STAT3, and EGFR. These proteins were selected because they occupied central positions within the PPI network and are recognized as major regulators of inflammatory signaling.

Molecular docking was employed to evaluate the potential binding affinity of the major phytochemicals identified in *S. peruviana* toward these biologically relevant targets. Prior to docking, the reliability of the docking protocol was validated by redocking the cocrystallized ligands into their respective binding pockets. In all cases, the obtained RMSD values were below 2.0 Å, indicating that the docking protocol was able to reproduce the experimentally observed binding mode of the cocrystallized ligands with acceptable accuracy.

Among the investigated phytochemicals, linoleic acid and α‐linolenic acid exhibited the highest predicted binding affinities toward TNF‐α (−6.1 kcal/mol), followed by hexahydrofarnesyl acetone (−6.0 kcal/mol), stearic acid (−5.8 kcal/mol), and palmitic acid (−5.6 kcal/mol), whereas the cocrystallized ligand displayed a binding energy of −7.4 kcal/mol. Although the phytochemicals showed lower predicted affinities than the native inhibitor, they occupied the same binding region and established interactions with several residues involved in ligand recognition, including TYR59, LYS98, ILE118, TYR119, LEU120, GLY121, and TYR151 (Table 3).

**TABLE 3:** Molecular docking results of the major *Scilla peruviana* L. phytochemicals against tumor necrosis factor‐alpha (TNF‐α, PDB ID: 2AZ5).

		Binding energy, kcal/mol	**Hydrogen interactions (distance Å)**	Hydrophobic interactions	Electrostatic interaction
Cocrystallized ligand	307	−7.4	GLY121 (2.76), GLY121 (2.98, C─H bond), GLN61 (2.81, C─H bond), GLY121 (2.91, C─H bond)	LEU57 (alkyl), TYR59 (π–alkyl), TYR119 (π–alkyl), TYR151 (π–alkyl), 3071 (π–π stacked ×3)	TYR119 (π–cation)
Best docked compounds	Hexaydrofarnesyl acetone	−6.0	GLY121 (2.42), LEU120 (2.55, C─H bond)	TYR59 (π–alkyl ×2), TYR151 (π–alkyl)	—
	Palmitic acid	−5.6	LYS98 (1.92), TYR119 (2.43), LYS98 (2.52, C─H bond), ILE118 (2.66, C─H bond)	TYR59 (π–alkyl), TYR151 (π–alkyl)	LYS98 (attractive charge)
	Linoleic acid	−6.1	LYS98 (2.00), TYR119 (2.11), LYS98 (2.73, C─H bond), ILE118 (2.60, C─H bond)	TYR119 (π–alkyl ×3)	LYS98 (attractive charge/salt bridge)
	Stearic acid	−5.8	LYS98 (1.96), LYS98 (2.72, C─H bond)	ALA96 (alkyl)	LYS98 (attractive charge ×2/salt bridge)
	Alpha‐linolenic acid	−6.1	LYS98 (2.81), LYS98 (2.30)	TYR119 (π–alkyl)	LYS98 (attractive charge/salt bridge)

Notably, linoleic acid and α‐linolenic acid formed hydrogen bonds and electrostatic interactions with LYS98, together with hydrophobic contacts involving TYR119, while hexahydrofarnesyl acetone interacted with GLY121, LEU120, TYR59, and TYR151, residues also contributing to stabilization of the cocrystallized inhibitor (Figure 8). Although the investigated phytochemicals established interactions within the TNF‐α binding pocket, their predicted binding energies were weaker than those of the cocrystallized ligand (−7.4 kcal/mol). These results therefore indicate potential ligand–protein interactions but do not provide evidence of stronger binding or TNF‐α inhibition. The observed interaction patterns should consequently be considered computationally predicted binding modes requiring experimental validation.

**FIGURE 8:**
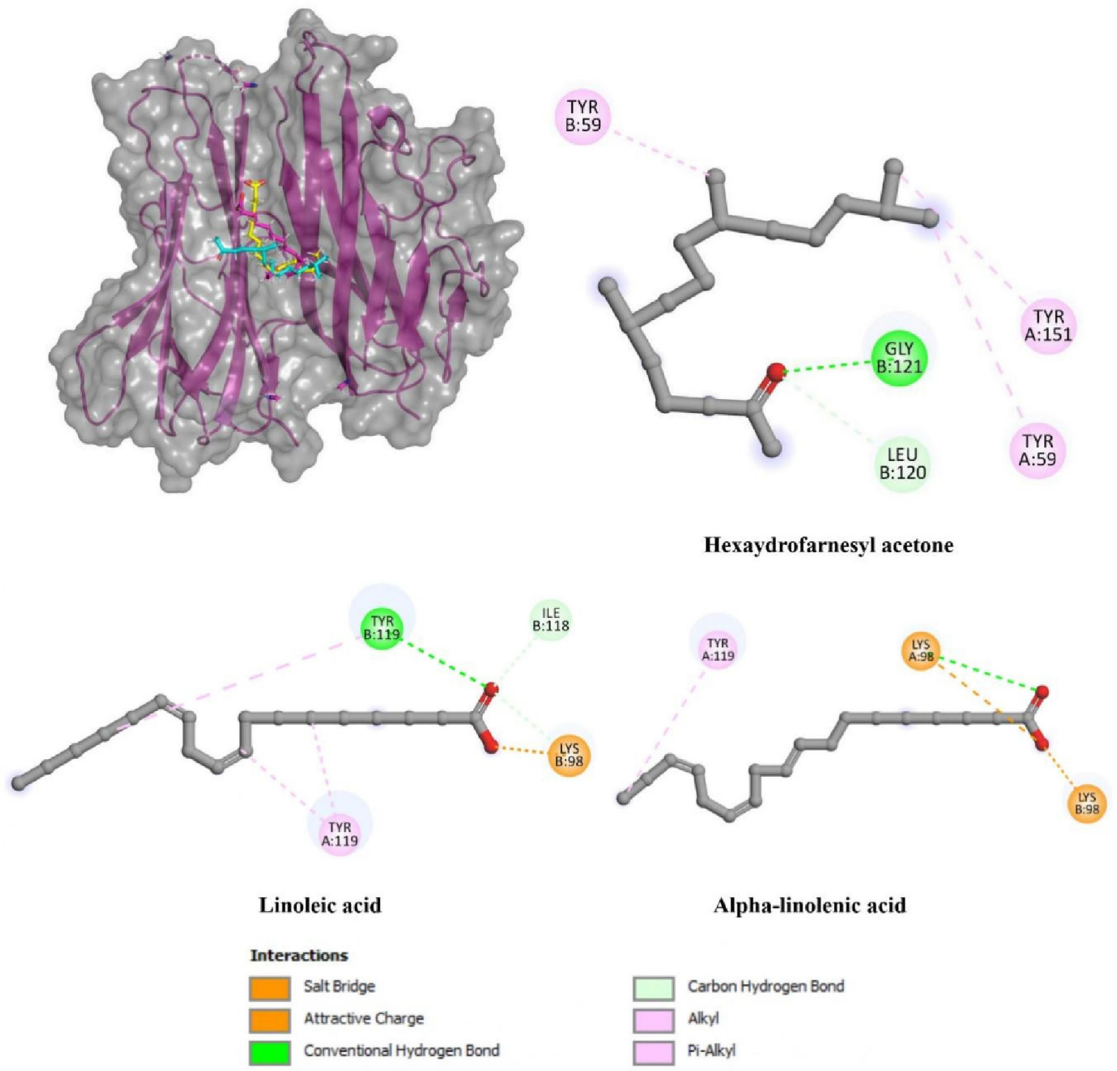
Molecular docking poses of the three best‐scoring *Scilla peruviana* phytochemicals within the binding pocket of tumor necrosis factor‐alpha (TNF‐α, PDB ID: 2AZ5). The figure illustrates the three‐dimensional binding conformations and the corresponding two‐dimensional interaction maps of hexahydrofarnesyl acetone, linoleic acid, and α‐linolenic acid, highlighting the principal hydrogen‐bonding, hydrophobic, electrostatic, and π‐mediated interactions with the binding‐site residues.

The docking analysis revealed that several *S. peruviana* phytochemicals exhibited relatively low predicted binding energies toward the COX‐2 active site. Among them, palmitic acid displayed the strongest predicted affinity (−7.0 kcal/mol), followed by stearic acid (−6.7 kcal/mol), α‐linolenic acid (−6.6 kcal/mol), linoleic acid (−6.1 kcal/mol), and hexahydrofarnesyl acetone (−5.8 kcal/mol). The cocrystallized ligand exhibited a binding energy of −6.4 kcal/mol (Table 4).

**TABLE 4:** Molecular docking results of the major *Scilla peruviana* L. phytochemicals against cyclooxygenase‐2 (COX‐2/PTGS2, PDB ID: 5IKR).

		Binding energy, kcal/mol	**Hydrogen interactions (distance Å)**	Hydrophobic interactions	Electrostatic interaction
Cocrystallized ligand	ID8	−6.4	TYR385 (1.53), SER530 (1.58)	ALA527 (alkyl ×2), VAL349 (alkyl ×3), LEU531 (alkyl ×2), VAL116 (alkyl), LEU352 (π–alkyl)	—
Best docked compounds	Hexaydrofarnesyl acetone	−5.8	—	VAL349 (alkyl ×2), ALA527 (alkyl ×4), LEU384 (alkyl), MET522 (alkyl), LEU352 (alkyl), LEU531 (alkyl ×2), VAL116 (alkyl), ARG120 (alkyl), TYR355 (π–alkyl), PHE381 (π–alkyl), TYR385 (π–alkyl), TRP387 (π–alkyl ×3), PHE518 (π–alkyl)	—
	Palmitic acid	−7.0	ARG120 (2.80, C─H bond)	VAL89 (alkyl), VAL116 (alkyl ×2), ARG120 (alkyl), VAL349 (alkyl ×2), ALA527 (alkyl ×2), MET522 (alkyl), LEU352 (alkyl), LEU531 (alkyl), TYR355 (π–alkyl), TRP387 (π–alkyl), PHE518 (π–alkyl)	ARG120 (attractive charge)
	Linoleic acid	−6.1	—	VAL116 (alkyl), VAL349 (alkyl ×2), VAL523 (alkyl), ALA527 (alkyl ×3), VAL344 (alkyl), LEU534 (alkyl), LEU352 (alkyl ×3), PHE205 (π–alkyl), TYR348 (π–alkyl), TYR355 (π–alkyl), TYR385 (π–alkyl)	ARG120 (attractive charge ×2)
	Stearic acid	−6.7	TYR115 (2.88), LYS83 (2.24, C─H bond)	VAL116 (alkyl ×2), VAL349 (alkyl ×2), ALA527 (alkyl ×3), LEU384 (alkyl), LEU352 (alkyl), LEU531 (alkyl), TYR355 (π–alkyl), TYR385 (π–alkyl), TRP387 (π–alkyl)	LYS83 (attractive charge)
	Alpha‐linolenic acid	−6.6	—	VAL116 (alkyl), VAL349 (alkyl ×2), ALA527 (alkyl ×3), LEU352 (alkyl ×3), VAL523 (alkyl), LEU384 (alkyl), TYR355 (π–alkyl), TYR385 (π–alkyl), TRP387 (π–alkyl), PHE518 (π–alkyl)	ARG120 (attractive charge ×2)
	3‐Ethyl‐4‐methyl‐1H‐pyrrole‐2,5‐dione	−5.3	TYR385 (2.00), SER530 (2.81), SER530 (2.66, C─H bond)	ALA527 (alkyl ×2), VAL349 (alkyl), LEU352 (alkyl), VAL523 (alkyl), LEU352 (π–alkyl)	—
	2‐Phenyl‐5‐methyl2‐hexenal	−5.9	SER353 (2.82, C─H bond)	GLY526/ALA527 (amide–π), ALA527 (alkyl), VAL116 (alkyl ×2), ARG120 (alkyl), LEU531 (alkyl), VAL349 (alkyl), LEU359 (alkyl), LEU352 (π–alkyl), ALA527 (π–alkyl)	—
	Megastigmatrienone	−5.5	—	LEU352 (alkyl ×2), VAL523 (alkyl), ALA527 (alkyl ×3), VAL349 (alkyl), LEU531 (alkyl), VAL523 (alkyl ×2), TYR355 (π–alkyl ×2), TYR385 (π–alkyl), TRP387 (π–alkyl)	—

Palmitic acid and stearic acid showed relatively low predicted binding energies toward COX‐2 (−7.0 and −6.7 kcal/mol, respectively), with palmitic acid showing a slightly lower predicted value than the cocrystallized ligand (−6.4 kcal/mol). These results suggest potentially favorable computational interactions within the COX‐2 binding pocket. However, docking scores alone cannot establish COX‐2 inhibition or anti‐inflammatory efficacy, and the biological significance of these interactions requires experimental confirmation. These compounds occupied the canonical COX‐2 binding pocket and established multiple hydrophobic interactions with residues lining the catalytic cavity, including VAL116, VAL349, LEU352, TYR355, TYR385, ALA527, and LEU531. Moreover, palmitic acid formed an electrostatic interaction with ARG120, a residue widely recognized as an important anchoring site for numerous COX inhibitors (Figure 9). The interaction profiles suggest that these phytochemicals are structurally compatible with the COX‐2 active site and may contribute to the anti‐inflammatory activity predicted by the network pharmacology analysis.

**FIGURE 9:**
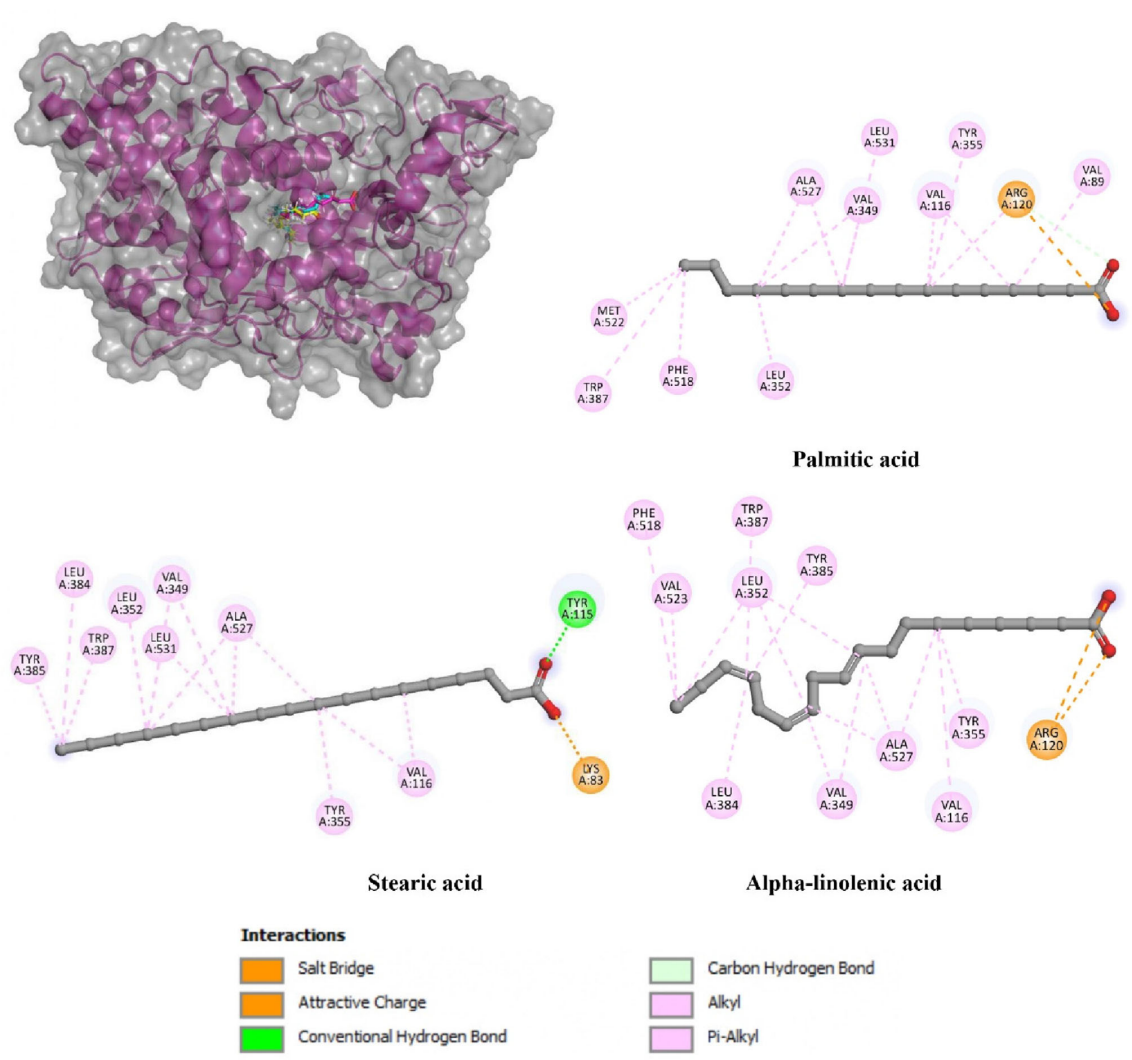
Molecular docking poses of the three best‐scoring *Scilla peruviana* L. phytochemicals within the catalytic pocket of cyclooxygenase‐2 (COX‐2/PTGS2, PDB ID: 5IKR). The figure presents the three‐dimensional binding conformations and the corresponding two‐dimensional interaction maps of palmitic acid, stearic acid, and α‐linolenic acid, illustrating the principal hydrogen‐bonding, hydrophobic, electrostatic, and π‐mediated interactions with the active‐site residues.

Molecular docking predicted relatively low binding energies for several investigated phytochemicals toward the PPARγ ligand‐binding domain. Hexahydrofarnesyl acetone and stearic acid showed the lowest predicted binding energies (−8.3 kcal/mol), approaching that of the cocrystallized agonist rosiglitazone (−8.6 kcal/mol). These results suggest that these compounds may adopt binding orientations compatible with the PPARγ ligand‐binding pocket; however, the docking results do not establish PPARγ agonism or anti‐inflammatory activity. Hexahydrofarnesyl acetone and stearic acid displayed the highest predicted binding energies (−8.3 kcal/mol), approaching that of the cocrystallized agonist rosiglitazone (−8.6 kcal/mol), whereas α‐linolenic acid, palmitic acid, and linoleic acid exhibited binding energies ranging from −7.6 to −7.9 kcal/mol (Table 5).

**TABLE 5:** Molecular docking results of the major *Scilla peruviana* L. phytochemicals against peroxisome proliferator‐activated receptor gamma (PPARγ, PDB ID: 4EMA).

		Binding energy, kcal/mol	**Hydrogen interactions (** **distance Å)**	Hydrophobic interactions	Electrostatic interaction
Cocrystallized ligand	BRL	−8.6	SER289 (1.66), HIS323 (2.32), HIS449 (1.92), TYR473 (1.73), CYS285 (2.57), HIS323 (2.43, C─H bond), ILE281 (2.46, C─H bond)	CYS285 (alkyl), VAL339 (alkyl), MET348 (alkyl), LEU353 (alkyl), MET364 (alkyl), CYS285 (π–alkyl ×2), ILE341 (π–alkyl), MET348 (π–alkyl), LEU330 (π–alkyl)	HIS323 (attractive charge)
Best docked compounds	Hexaydrofarnesyl acetone	−8.3	TYR473 (1.98), HIS323 (2.66, C─H bond)	CYS285 (alkyl ×2), ILE281 (alkyl), MET348 (alkyl ×2), ILE341 (alkyl), ARG288 (alkyl), LEU330 (alkyl ×2), MET364 (alkyl ×2), TYR327 (π–alkyl), PHE363 (π–alkyl), HIS449 (π–alkyl)	—
	Palmitic acid	−7.6	HIS323 (2.07), TYR473 (2.08), HIS323 (2.53, C─H bond)	CYS285 (alkyl ×3), ARG288 (alkyl ×2), ILE341 (alkyl ×2), LEU330 (alkyl), MET364 (alkyl), TYR327 (π–alkyl)	HIS323 (attractive charge)
	Linoleic acid	−7.6	SER289 (2.16), HIS449 (2.63), HIS323 (2.45, C─H bond)	CYS285 (alkyl ×4), ARG288 (alkyl ×2), VAL339 (alkyl ×2), ILE341 (alkyl ×2), MET348 (alkyl), LEU330 (alkyl ×2)	HIS323 (attractive charge)
	Stearic acid	−8.3	SER289 (2.15), HIS449 (2.54), TYR473 (1.89), HIS323 (2.46, C─H bond)	CYS285 (alkyl ×3), ARG288 (alkyl ×2), LEU255 (alkyl), ARG280 (alkyl), ILE281 (alkyl), ILE341 (alkyl ×2), MET348 (alkyl), LEU330 (alkyl ×2), ILE326 (alkyl)	HIS323 (attractive charge/salt bridge)
	Alpha‐linolenic acid	−7.9	HIS449 (2.09), HIS323 (2.65, C─H bond)	CYS285 (alkyl ×4), VAL339 (alkyl), ILE341 (alkyl ×3), MET348 (alkyl ×2), LEU330 (alkyl ×2), MET364 (alkyl), ILE326 (alkyl)	HIS323 (attractive charge)
	2‐Phenyl‐5‐methyl2‐hexenal	−6.0	LYS367 (2.17, C─H bond), HIS449 (2.67, C─H bond)	LEU469 (alkyl), HIS323 (π–alkyl), TYR327 (π–alkyl), HIS449 (π–alkyl ×2), CYS285 (π–alkyl), ARG288 (π–alkyl), LEU330 (π–alkyl)	—

Analysis of the binding modes revealed that the docked phytochemicals occupied the same ligand‐binding cavity as rosiglitazone and interacted with several residues essential for ligand stabilization, including CYS285, HIS323, TYR473, HIS449, MET348, LEU330, ILE341, and MET364. In particular, hydrogen bonding with HIS323, SER289, HIS449, or TYR473, together with extensive hydrophobic contacts involving the surrounding residues, contributed to stabilization of the docked complexes (Figure 10). The conservation of these key interactions supports the structural compatibility of the identified phytochemicals with the PPARγ binding pocket.

**FIGURE 10:**
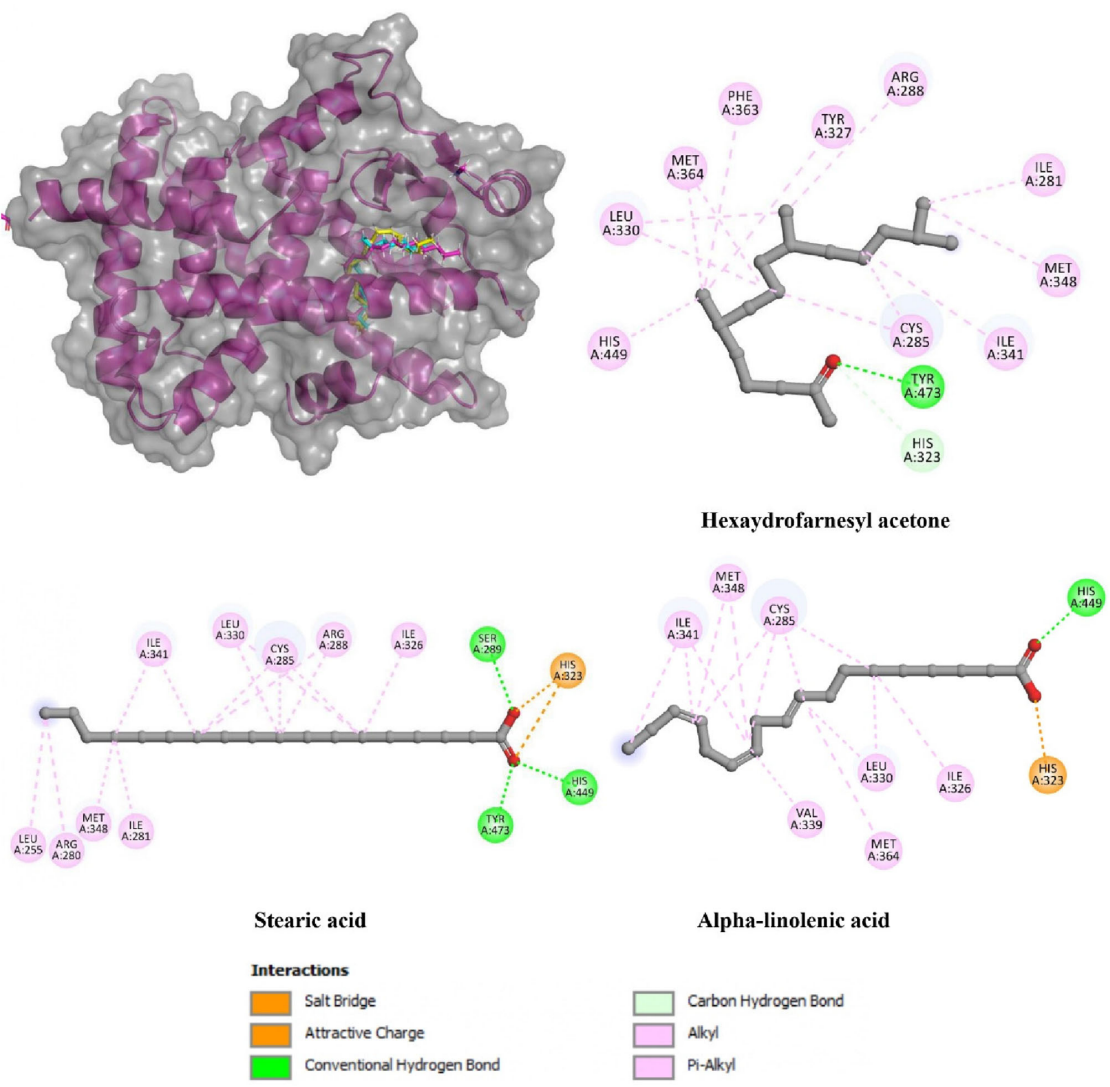
Molecular docking poses of the three best‐scoring *Scilla peruviana* phytochemicals within the ligand‐binding domain of peroxisome proliferator‐activated receptor gamma (PPARγ, PDB ID: 4EMA). The figure shows the three‐dimensional binding conformations and the corresponding two‐dimensional interaction maps of hexahydrofarnesyl acetone, stearic acid, and α‐linolenic acid, highlighting the principal predicted intermolecular interactions observed in the docked complexes.

Among the evaluated phytochemicals docked against STAT3 (PDB ID: 6NJS), linoleic acid exhibited the highest predicted binding affinity (−6.6 kcal/mol), followed by stearic acid and α‐linolenic acid (−6.3 kcal/mol), hexahydrofarnesyl acetone (−5.2 kcal/mol), and palmitic acid (−5.0 kcal/mol). As expected, the cocrystallized ligand SD36 displayed a substantially stronger binding affinity (−10.7 kcal/mol) (Table 6). The substantially weaker predicted binding energies of the investigated phytochemicals compared with the cocrystallized ligand indicate that their predicted interactions with STAT3 are less favorable under the applied docking conditions. Therefore, these results should be interpreted cautiously and should not be considered evidence of STAT3 inhibition.

**TABLE 6:** Molecular docking results of the major *Scilla peruviana* L. phytochemicals against signal transducer and activator of transcription 3 (STAT3, PDB ID: 6NJS).

		Binding energy, kcal/mol	**Hydrogen interactions (** **distance Å)**	Hydrophobic interactions	Electrostatic interaction
Cocrystallized ligand	SD36	−10.7	ARG609 (2.03), ARG609 (1.72), SER611 (1.53), GLU612 (1.89), SER613 (1.84, 1.54), GLU638 (1.79), GLN644 (1.77), TYR657 (1.74), SER636 (1.93), GLU638 (1.87), GLU638 (1.87), PRO639 (2.82), GLN644 (2.54), SER611 (2.81, C─H bond), SER611 (2.99, C─H bond), VAL637 (2.55, C─H bond)	VAL637/GLU638 (amide–π ×2), PRO639 (π–alkyl ×2)	LYS591 (attractive charge/salt bridge), ARG609 (attractive charge ×2)
Best D docked compounds	Hexaydrofarnesyl acetone	−5.2	ARG609 (1.96), GLU612 (2.13), LYS591 (2.88, C─H bond), LYS591 (3.09, C─H bond)	LYS591 (alkyl), PRO639 (alkyl)	—
	Palmitic acid	−5.0	LYS557 (1.97), LYS591 (2.68, C─H bond)	PRO639 (alkyl)	LYS591 (attractive charge/salt bridge)
	Linoleic acid	−6.6	ARG609 (2.01), SER611 (1.79), GLU612 (2.21), SER613 (2.04, 1.92), LYS591 (2.71, C─H bond), LYS591 (2.78, C─H bond), SER611 (3.06, C─H bond)	VAL637 (alkyl), ILE659 (alkyl ×2)	LYS591 (attractive charge/salt bridge)
	Stearic acid	−6.3	LYS591 (1.86), SER611 (1.71), GLU612 (2.26), SER613 (2.00, 1.82), SER611 (3.08, C─H bond)	PRO639 (alkyl), ILE659 (alkyl)	LYS591 (attractive charge/salt bridge), ARG609 (attractive charge ×2)
	Alpha‐linolenic acid	−6.3	LYS591 (1.89), SER611 (1.80), GLU612 (2.22), SER613 (2.12, 1.98), LYS591 (2.96, C─H bond), SER611 (3.01, C─H bond)	ILE659 (alkyl)	LYS591 (attractive charge), ARG609 (attractive charge)

Despite their lower binding energies, the phytochemicals established interactions with several residues involved in ligand recognition, including LYS591, ARG609, SER611, GLU612, SER613, PRO639, VAL637, and ILE659. In particular, linoleic acid, stearic acid, and α‐linolenic acid reproduced several hydrogen‐bonding interactions observed for the native ligand while simultaneously forming electrostatic interactions with LYS591 and ARG609 (Figure 11). These findings suggest that the investigated compounds are capable of interacting with functionally relevant regions of STAT3, although with a lower predicted affinity than the reference inhibitor.

**FIGURE 11:**
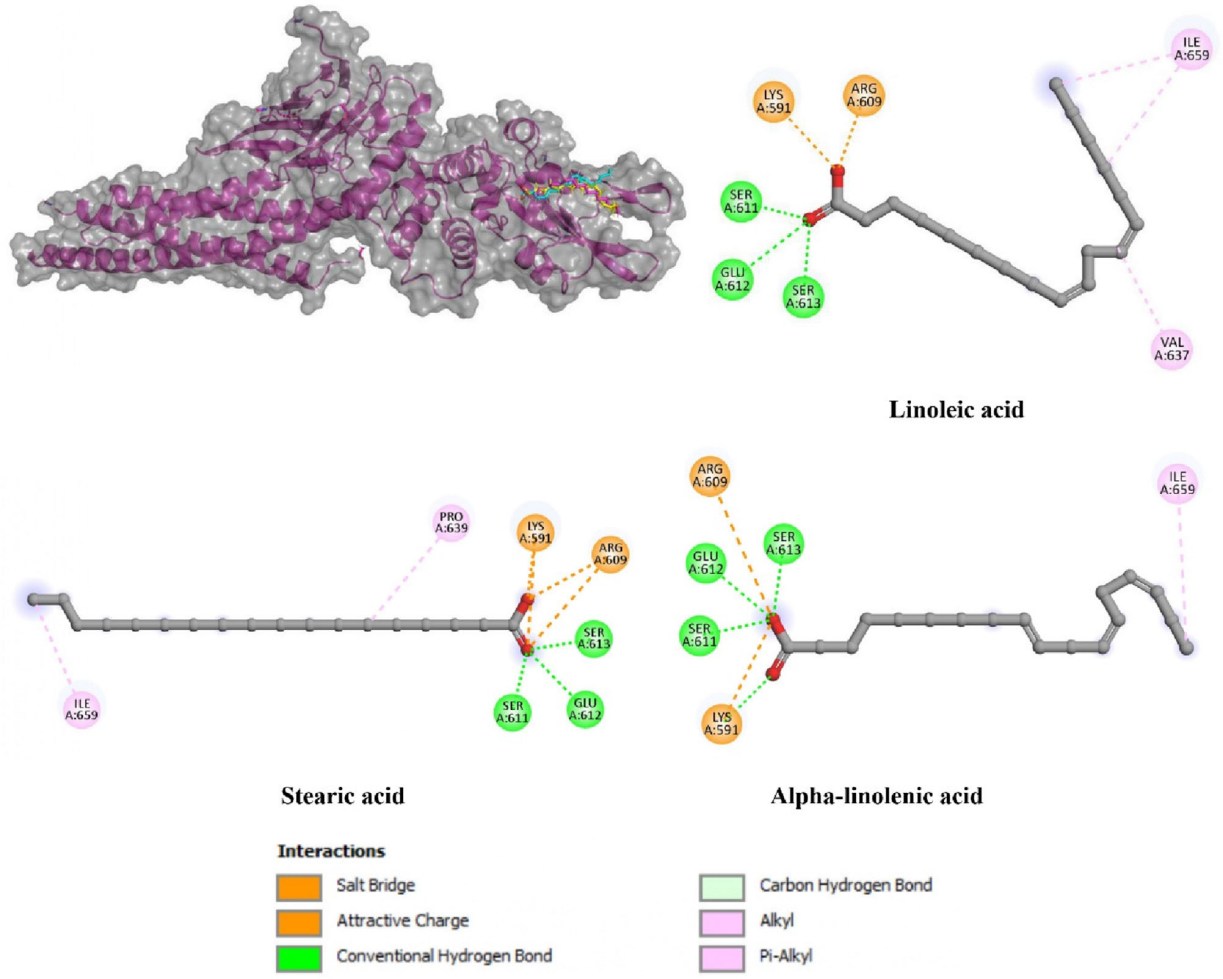
Molecular docking poses of the three best‐scoring *Scilla peruviana* L. phytochemicals within the binding pocket of signal transducer and activator of transcription 3 (STAT3, PDB ID: 6NJS). The figure displays the three‐dimensional binding conformations and the corresponding two‐dimensional interaction maps of linoleic acid, stearic acid, and α‐linolenic acid, emphasizing the principal hydrogen‐bonding, hydrophobic, electrostatic, and π‐mediated interactions with the binding‐site residues.

For EGFR (PDB ID: 1M17), molecular docking indicated that palmitic acid exhibited the strongest predicted binding affinity toward EGFR (−6.8 kcal/mol), followed by stearic acid (−6.6 kcal/mol), linoleic acid and α‐linolenic acid (−6.4 kcal/mol), and hexahydrofarnesyl acetone (−6.1 kcal/mol). The cocrystallized inhibitor erlotinib displayed a binding energy of −7.5 kcal/mol (Table 7).

**TABLE 7:** Molecular docking results of the major *Scilla peruviana* L. phytochemicals against epidermal growth factor receptor (EGFR, PDB ID: 1M17).

		Binding energy, kcal/mol	**Hydrogen interactions (distance Å)**	Hydrophobic interactions	Electrostatic interaction
Cocrystallized ligand	AQ4	−7.5	MET769 (2.13), GLY772 (2.47, C─H bond), GLN767 (2.30, C─H bond), AQ4999:O1 (2.58, C─H bond)	LYS721 (alkyl ×2), LEU764 (alkyl), ALA719 (π–alkyl), LEU820 (π–alkyl ×2), LEU694 (π–alkyl), VAL702 (π–alkyl), ALA719 (π–alkyl)	—
Best D docked compounds	Hexaydrofarnesyl acetone	−6.1	—	VAL702 (alkyl), ALA719 (alkyl ×3), LYS721 (alkyl ×2), LEU820 (alkyl ×2), LEU768 (alkyl), MET769 (alkyl), LEU694 (alkyl)	—
	Palmitic acid	−6.8	LYS704 (2.07), LYS704 (2.84, C─H bond)	VAL702 (alkyl), ALA719 (alkyl ×2), LYS721 (alkyl), MET742 (alkyl), LEU820 (alkyl ×2), LEU694 (alkyl ×3), LEU768 (alkyl)	LYS704 (attractive charge/salt bridge)
	Linoleic acid	−6.4	THR766 (2.06)	VAL702 (alkyl), ALA719 (alkyl), LEU694 (alkyl ×4), LEU820 (alkyl)	LYS721 (attractive charge)
	Stearic acid	−6.6	LYS692 (2.32), LYS692 (2.50, C─H bond), LYS704 (2.23, C─H bond)	VAL702 (alkyl ×2), ALA719 (alkyl ×2), LYS721 (alkyl ×2), MET742 (alkyl), LEU694 (alkyl ×3), LEU820 (alkyl), LEU768 (alkyl)	LYS692 (attractive charge), LYS704 (attractive charge)
	Alpha‐linolenic acid	−6.4	LYS692 (2.66, C─H bond), LYS704 (2.23, C─H bond)	VAL702 (alkyl ×2), LYS721 (alkyl ×2), MET742 (alkyl), LEU764 (alkyl), LEU820 (alkyl ×2), LEU694 (alkyl ×2)	LYS692 (attractive charge), LYS704 (attractive charge)

The docked phytochemicals occupied the ATP‐binding pocket of EGFR and established predominantly hydrophobic interactions with residues such as VAL702, ALA719, LEU694, LEU768, LEU820, MET742, and LYS721, together with hydrogen bond and electrostatic interactions involving LYS692, LYS704, or THR766 (Figure 12). Several of these residues are also involved in stabilization of the cocrystallized inhibitor, suggesting that the phytochemicals adopt binding orientations compatible with the native ligand‐binding site. These interaction patterns are consistent with the computational prediction of EGFR association obtained from network pharmacology analysis, but they do not establish EGFR inhibition or a direct anti‐inflammatory effect.

**FIGURE 12:**
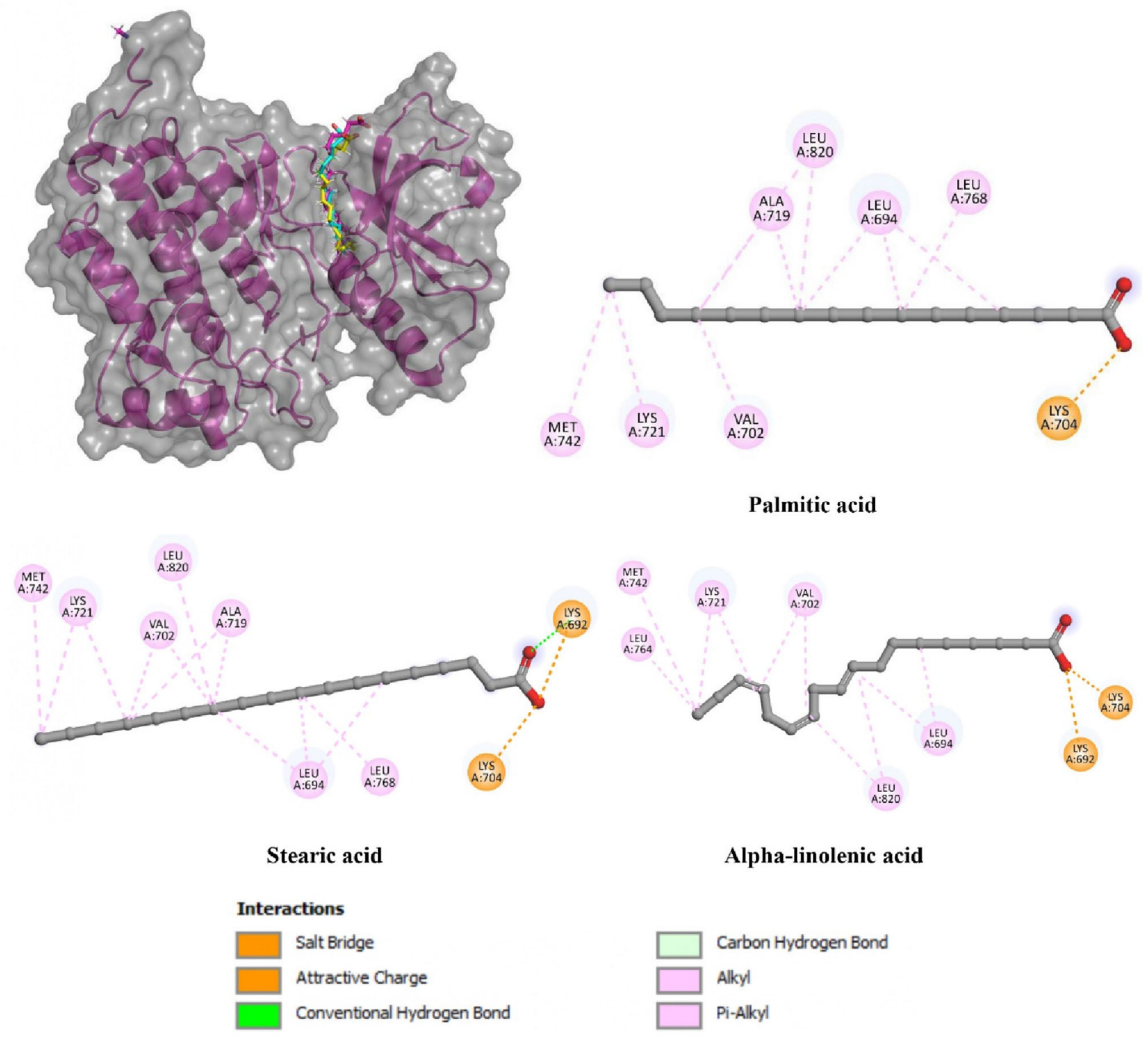
Molecular docking poses of the three best‐scoring *Scilla peruviana* L. phytochemicals within the ATP‐binding pocket of epidermal growth factor receptor (EGFR, PDB ID: 1M17). The figure presents the three‐dimensional binding conformations and the corresponding two‐dimensional interaction maps of palmitic acid, stearic acid, and α‐linolenic acid, illustrating the principal hydrogen‐bonding, hydrophobic, electrostatic, and π‐mediated interactions with the active‐site residues.

## 4 Conclusions

This study provides an integrated computational investigation of the potential anti‐inflammatory relevance of the volatile phytochemicals identified in *Scilla peruviana* L. By combining GC–MS characterization with network pharmacology, functional enrichment, and molecular docking, the study identified inflammation‐associated targets and signaling pathways and explored potential interactions between selected phytochemicals and key hub proteins, including TNF‐α, COX‐2, PPARγ, STAT3, and EGFR.

The network pharmacology analysis identified 253 overlapping targets and highlighted TNF, IL1B, EGFR, PTGS2, STAT3, and PPARG among the highly connected proteins. Functional enrichment further indicated associations with several inflammation‐related BP and signaling pathways, including NF‐κB, IL‐17, NOD‐like receptor, and AGE–RAGE signaling. Molecular docking provided computational evidence of potential interactions between several identified constituents and the selected protein targets, with palmitic acid showing the lowest predicted binding energy toward COX‐2 and hexahydrofarnesyl acetone and stearic acid showing relatively low predicted binding energies toward PPARγ.

However, these findings are based exclusively on computational predictions and should not be interpreted as experimental evidence of anti‐inflammatory activity. In particular, docking scores represent predicted ligand–protein binding affinities and do not establish target inhibition, receptor activation, or biological efficacy. Experimental validation using appropriate in vitro and in vivo models, together with pharmacokinetic and mechanistic investigations, is therefore required to determine whether the predicted molecular interactions translate into measurable biological effects. The present findings should consequently be considered hypothesis‐generating and may provide a basis for guiding future experimental studies on the biological potential of *S. peruviana* constituents.

## Author Contributions

**Roukia Zatout** **:** conceptualization, resources, methodology, investigation, writing – original draft, supervision, review, and editing. **Ouided Benslama**: formal analysis, data curation, software, writing – original draft. **Stefania Garzoli**: formal analysis, data curation, methodology, investigation, supervision, writing, review, and editing.

## Funding

The authors have nothing to report.

## Conflicts of Interest

The authors declare no conflicts of interest.

## Data Availability

The data that support the findings of this study are available from the corresponding author upon reasonable request.
